# Benefit of Contact Force Sensing Catheter Technology for Successful Left Atrial Anterior Line Formation: A Prospective Randomized Trial

**DOI:** 10.1155/2018/9784259

**Published:** 2018-09-18

**Authors:** Stephanie Fichtner, Reza Wakili, Konstantinos Rizas, Johannes Siebermair, Moritz F. Sinner, Thomas Wiktor, Korbinian Lackermair, Franziska Schuessler, Lucia Olesch, Susanne Rainer, Stefan Kääb, Adrian Curta, Harald Kramer, Heidi L. Estner

**Affiliations:** ^1^Department of Medicine I, University Hospital Munich, Ludwig-Maximilians University, Munich, Germany; ^2^German Cardiovascular Research Centre (DZHK), Partner Site: Munich Heart Alliance, Munich, Germany; ^3^Department of Cardiology and Vascular Medicine, West-German Heart and Vascular Center Essen, Essen University Hospital, University Duisburg-Essen, Essen, Germany; ^4^Charité Campus Virchow-Klinikum, Berlin, Germany; ^5^Institute for Clinical Radiology, University Hospital Munich, Ludwig-Maximilians University, Munich, Germany

## Abstract

**Introduction:**

The value of contact force information for ablation of LA anterior line is unknown. In a prospective randomized clinical trial, we investigated if information on contact force during left atrial (LA) anterior line ablation reduces total radiofrequency time and results in higher rates of bidirectional line block in patients undergoing pulmonary vein isolation (PVI) plus substrate modification.

**Methods:**

We included patients with indication for pulmonary vein isolation (PVI) and additional substrate modification. For LA anterior line ablation, patients were randomized to contact force information visible (n=35) or blinded (n=37). Patients received contrast enhanced cardiac magnetic resonance imaging (cMRI) before and 3-6 months after ablation to visualize the LA anterior line. Primary endpoint was radiofrequency time to achieve bidirectional line block. Secondary endpoints were completeness of the LA anterior line on cMRI, distribution of contact force, procedural data, adverse events, and 12 months success rate.

**Results:**

In 72 patients (64±9 years, 68% male), bidirectional LA anterior line block was achieved in 70 (97%) patients. Radiofrequency time to bidirectional block did not differ significantly across groups (contact force information visible 23±18min versus contact force information blinded 21±15min, p=0.50). The LA anterior line was discernable on cMRI in 40 patients (82%) without significant differences across randomization groups (p=0.46). No difference in applied contact force was found depending on cMRI line visibility. Twelve-month success and adverse event rates were comparable across groups.

**Conclusion:**

Information on contact force does not significantly improve the ablation of LA anterior lines.

**Clinical Trial Registration:**

The trial was registered at http://www.clinicaltrials.gov by identifier: NCT02217657.

## 1. Introduction

Catheter ablation of atrial fibrillation (AF) has evolved as a guideline recommended therapeutic option for patients with this condition. Whereas wide area circumferential pulmonary vein isolation (PVI) comprises the standard approach, selected patients require additional left atrial (LA) linear lesions [1]. By applying LA linear lesions, the goal is to achieve bidirectional conduction block, as incomplete block predisposes to the development of atypical atrial flutter. However, depending on the LA anatomy, bidirectional block of the anterior line can only be achieved in 60% to 86% [2, 3].

Preclinical research showed that appropriate contact force between the ablation catheter tip and the target tissue is a key contributor to effective lesion formation [4, 5]. Insufficient contact may result in an ineffective lesion, leading to arrhythmia recurrence, whereas excessive contact force may result in tissue damage and possibly life threatening complications [5]. In multicenter clinical trials, PVI using contact force sensing catheter technology resulted in a reduced rate of immediate, intraprocedural pulmonary vein reconnection [6], and reduced rates of AF recurrence during follow-up [7]. However, the benefit of using contact force sensing catheter technology is unclear for the formation of LA linear lesions.

Thus, in our study, we included patients with persistent AF or AF recurrence after a first PVI and randomized them to receive a LA anterior line ablation using either visible or blinded contact force sensing information. Beyond measures of procedural success, we systematically obtained cardiac magnetic resonance imaging (cMRI) to visually assess the ablation success.

## 2. Methods

### 2.1. Study Cohort

Patients scheduled for catheter ablation of drug refractory persistent AF or for catheter reablation of AF recurrence after PVI for paroxysmal AF were eligible for study participation because of needed additional substrate modification. We excluded patients for the following reasons: left atrial thrombus, symptomatic mitral valve stenosis or moderate to severe mitral valve insufficiency (≥ grade 2 of 4), severely impaired left ventricular function (left ventricular ejection fraction ≤35%), hyperthyroidism, current pregnancy, and age <18 years or > 80 years. All patients provided written informed consent. The ethics committee at the Ludwig-Maximilians University, Munich, Germany, approved the study, which was also registered at www.clinicaltrials.gov (NCT02217657).

**Figure 1:**
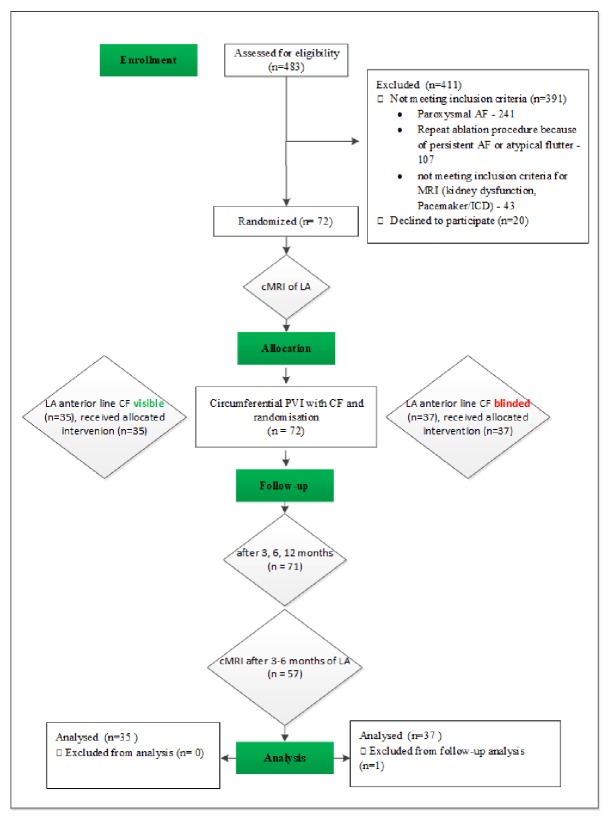
Study flowchart. Ext CV: external cardioversion; AF: atrial fibrillation; cMRI: cardiac magnet resonance imaging; LA: left atrium; CF: contact force; PVI: pulmonary vein isolation.

After written informed consent, through an investigator not involved in the ablation procedure, all enrolled patients received a wide area circumferential PVI and were intraprocedurally randomized in a 1:1 fashion to receive a left atrial anterior line either with or without real-time display of catheter tip-to-tissue contact force sensing information to the investigator. Randomization was undertaken using sealed envelopes.  Figure 1 summarizes the study procedures.

Our primary endpoint was the radiofrequency time to achieve bidirectional block of LA anterior line. Secondary endpoints were procedural duration, fluoroscopy time, and radiation dose and differences in contact force measures between both groups. We further visually assessed LA anterior line lesion formation by cMRI. Over the follow-up duration of 12 months, we also compared the occurrence of adverse events and the postprocedural atrial arrhythmia recurrence rate.

### 2.2. Ablation Procedure

Patients were kept on continuous oral anticoagulation, aiming for an international normalized ratio (INR) of 2.0-2.7. For patients using Dabigatran, Apixaban, or Rivaroxaban, the last dose was paused. Ablation procedures were performed under conscious sedation with midazolam and remifentanil. For electroanatomical mapping and three-dimensional navigation, we used the Carto 3 mapping system (Biosense Webster Inc., Diamond Bar, CA, USA). A 10-electrode deflectable catheter was placed in the coronary sinus (Inquiry, St. Jude Medical, LLC, St. Paul, MN, USA), and the aortic root was marked using a pigtail catheter introduced via the radial artery. The LA was accessed by single or double transseptal puncture via a steerable sheath (Agilis, St. Jude Medical, LLC, St. Paul, MN, USA). A fluoroscopic angiogram of all pulmonary veins was performed prior to the ablation. During the LA procedure time, intensified anticoagulation was maintained with unfractionated heparin aiming for an activated clotting time of ≥300 seconds.

For PVI, a steerable 20 electrode circular mapping catheter (Lasso, Biosense Webster Inc., Diamond Bar, CA, USA) was placed inside the pulmonary veins via the steerable sheath. Radiofrequency (RF) ablation was then performed using an open irrigated tip ablation catheter with contact force sensing capabilities (Smart Touch, Biosense Webster Inc., Diamond Bar, CA, USA), mostly without usage of a sheath. Catheter settings were as follows: 30 watts, power controlled with a temperature maximum of 43° Celsius, and an irrigation rate of 30 ml/min. We aimed for an ipsilateral wide antral circumferential isolation of all pulmonary veins in all patients regardless if PVI was performed previously. RF lesions were done using the dragging method between ablation points. Main target for individual lesion was electrogram diminution with a contact force above 10 g. Pulmonary vein isolation was confirmed by documentation of entrance and exit block interpreting the bipolar electrograms of the circular mapping catheter.

Following successful PVI, all patients received a LA anterior line, depending on randomization using visible or blinded contact force sensing information, since 10/2013 Visitag was available and not displayed in the blinded group. The LA anterior line was formed using the same RF ablation catheter as for PVI, connecting the anterior mitral annulus with the left superior pulmonary. LA anterior line formation was considered successful by documentation of bidirectional block using differential pacing criteria and by documentation of double potentials along the line [3, 8]. In the group with contact force displayed, real-time contact force was visible throughout the procedure, and investigators aimed for 10-30g [9]. In the contact force blinded group, investigators relied on standard criteria for ablation guidance. No other line apart from LA anterior line was performed.

After a waiting period of 30 minutes after successful formation of the LA anterior line, PVI isolation was reconfirmed including fractionated application of intravenous adenosine for each PV to rule out dormant conduction [10]. At the LA anterior line, bidirectional conduction block was reconfirmed.

### 2.3. Cardiac Magnetic Resonance Imaging

cMRI was performed with a 3-Tesla system (Magnetom Verio, Siemens Healthineers, Erlangen, Germany). A first cMRI was performed prior to the ablation procedure to visualize preexisting scar tissue in the ablation region. To improve image quality, patients in AF underwent external cardioversion prior to the scan. A second cMRI was scheduled three to six months after the ablation for visualization of the anterior line.

Methodologically, fast low angle shot-inversion recovery sequences (FLASH-IR) were performed 15 minutes after contrast agent application (Gadobutrol 0.15mmol/kgbw, Gadovist, BayerSchering, Berlin, Germany) (ST=0,9mm, TE=1.460ms, TR=462.560ms, FA=20°) for analysis of ablation lesions by late gadolinium enhancement (LGE). Two investigators blinded to randomization status and clinical parameters independently assessed the LA anterior line visually comparing both available cMRI scans using the Siemens Argus Software (Siemens Healthineers, Erlangen, Germany). In case of discrepancies, a final judgment was reached by consent. LA anterior line assessment was standardized by defining three distinct line segments that were assessed individually: (a) anterior mitral valve annulus to inferior left atrial appendage; (b) septal of the left atrial appendage; (c) roof of the left atrial appendage to the left superior pulmonary vein (Figure 3). The same segment based analysis was performed for the secondary analysis of locally applied contact force data.

### 2.4. Follow-Up

Systematic follow-up was scheduled at the arrhythmia clinic 3, 6, and 12 months after the ablation procedure and done through physicians who were not part of the ablation team and were blinded to treatment group. At each visit, arrhythmia-related symptoms and adverse events were surveyed. A 7-day Holter-ECG was obtained at each visit. For AF recurrence analysis, we considered a blanking period of 3 months. After this period, AF recurrence was defined as any documented atrial arrhythmia of ≥30 second's duration. [1] Because the subgroup of patients with repeat ablation due to relapse of paroxysmal AF was too small, only patients with persistent AF and first ablation procedure were analyzed regarding sinus rhythm after 12 months. Oral anticoagulation was discontinued in patients without AF recurrence after 6 months in case of a CHA_2_DS_2_-VASc score <2.

### 2.5. Statistical Analysis

We based our power calculation on the experience of a previous study [3]. We assumed a RF application time of 17 minutes for LA anterior line formation without information on contact force. With contact force information available, we expected a 40% reduction of RF application time (10 minutes) [11]. Aiming for a two-sided *α* of 5% and a power of 80%, we calculated a sample size of 62 patients (31 patients per group). To account for a primary success rate of LA anterior line conduction block of 86%, we planned our study to enroll 72 patients (36 patients per group).

Statistical analysis was performed using SPSS (version 24, IBM SPSS Statistics, IBM Corp, Amonk, NY) and R studio (R foundation for statistical computing, Vienna Austria). Continuous variables are presented as mean ± standard deviation and are compared using the Wilcoxon rank sum or Student's* t*-tests. Categorical data are presented as frequency and percentages and are analyzed using the chi-square or Fisher exact tests. In addition, we fitted linear regression models to account for potential confounding by age, sex, and CHA2DS2-VASc score. P values <0.05 were considered statistically significant.

## 3. Results

### 3.1. Patient Characteristics

Between 2012 and 2015, 72 patients were prospectively randomized and included in the analysis. No significant differences in baseline characteristics were noted between both study groups (Table 1).

### 3.2. Procedural Results

**Figure 2:**
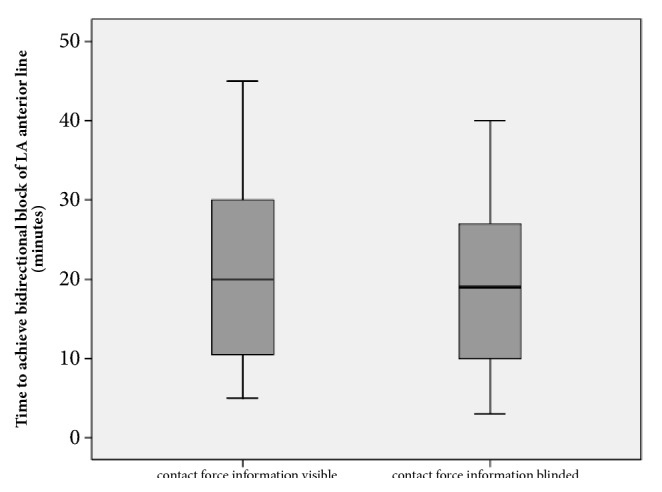
The primary endpoint of time to bidirectional block of the LA anterior line did not differ between both groups.

All patients received PVI and additional ablation of a LA anterior line as planned and according to randomization status. PVI was procedurally successfully in all patients (100%). In 70 of 72 patients (97%), a bidirectional block of the LA anterior line was achieved. The primary endpoint being radiofrequency time to achieve bidirectional block at the LA anterior line did not differ significantly (p=0.50) between both groups with 23±18min (contact force information visible) versus 21±15min (contact force information blinded) (Table 2; Figure 2).

In addition, secondary endpoints did not differ significantly between both groups: overall procedure time (contact force information visible: 192.6±30 min versus contact force information blinded: 189±60 min, p=0.70); overall fluoroscopy time (23.3±10 min versus 27.9±13min, p=0.12); overall radiation dose (1930±2025 cGy*∗*cm^2^ versus 1692±1128 cGy*∗*cm^2^, p=0.57). Details are provided in Table 2.

**Figure 3:**
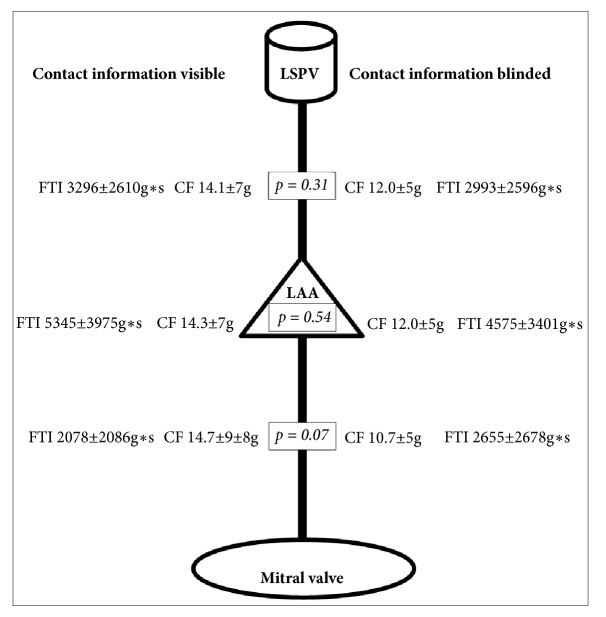
Distribution of contact force and force time integral in different segments of the anterior line. LAA: left atrial appendage; CF: contact force; FTI: force time integral; LSPV: left superior pulmonary vein. No significant difference could be detected between groups.

Analysis of contact force information and force time integral at the LA anterior line by prespecified line segments similarly revealed no significant differences between both groups (Figure 3). Across line segments, contact force >10g was achieved in 80% of applied lesions.

Six patients (16%) in the visible group and six patients (17%) in the blinded group showed a reconnected LA anterior line after the waiting period and were successfully reablated showing no significant difference between both groups. Patients with reconnection compared to patients without showed no significant difference in applied contact force (13.1±4g versus 13.6±6g respectively, p = 0.8).

The secondary endpoint comparing contact force in three distinct segments of the LA anterior line did not reveal significant differences across randomization groups: LSPV, left superior pulmonary vein; LAA, left atrial appendage; CF, contact force (Figure 3).

Multivariable adjusted analyses suggested no confounding by cMRI derived end-diastolic or end-systolic left ventricular volume, LA ejection fraction (cMRI), left ventricular ejection fraction (echocardiography), and ablation in sinus-rhythm or AF.

### 3.3. MRI Results

In 49 patients, both the cMRI acquired at baseline and at follow-up showed sufficient image quality for analysis. The remaining patients were not suitable for analysis due to insufficient image quality (n=8), incident pacemaker implantation after the baseline scan (n=4), and withdrawal of consent for the follow-up cMRI (n=11).

In the initial cMRI, LGE at the anterior wall was present in six patients (15%) without a difference between both groups (p=0.19). LGE before ablation procedure did not influence time to achieve bidirectional block of anterior line and did not influence outcome after 12-month follow-up. After the ablation, the LA anterior line was visible on cMRI in at least one segment in 40 patients (82%), regardless of whether contact force was displayed or not (p=0.46). Restricting the analysis to those patients with visible LA anterior line on cMRI, we similarly found no significant differences in contact force between randomization groups (contact force information visible: 12.9±4g versus contact force information blinded: 11.6±1.8g, p=0.42) (Figure 3).

In 26 patients (53%), which underwent a post-cMRI, a complete LA anterior line was detected by cMRI in all three segments, with no difference between both groups (p=0.78). In addition, no significant differences regarding contact force could be detected (contact force information visible: 14±7g versus contact force information blinded: 12.7±3g, p=0.31). Segment-specific analysis revealed that the LA anterior line was visible in 66% of patients between the anterior mitral annulus and the left atrial appendage, in 61% of patients septal of the left atrial appendage, and in 75% of patients between the left atrial appendage and the left superior pulmonary vein. For neither of the segments did we identify significant differences in contact force: contact force information visible: 14.7±9g, 14.3±7g, and 14.1±7g, respectively, versus 10.7±5g, 13.2±5g, and 12.0±5g, respectively; p=0.07, p=0.54, and p=0.31, respectively. Multivariable adjustment did not relevantly change these results.

### 3.4. Follow-Up at 12 Months and Repeat Ablation Procedures

Follow-up at 12 months was available for 71 (99%) patients, with one patient lost to follow-up. The recurrence rate of any documented atrial arrhythmia in patients with persistent AF off any antiarrhythmic class I or class III medication was 45% (contact force information visible) versus 52% (contact force information blinded) after a single procedure (p=0.8).

Overall, 17 patients underwent repeat ablation, equivalent to 23% of patients in the contact force information visible group and 29% of patients in the contact force information blinded group (p=0.8). The rates of left atrial flutter (contact force information visible: 37% versus contact force information blinded: 44%, p=0.16) and the percentages of sustained bidirectional block of the LA anterior line during the repeat procedure (contact force information visible: 37% versus contact force information blinded: 44%, p=0.1) did not differ significantly. In 10 of 17 patients with repeat ablation cMRI was available. Of these, eight patients showed a reconnected LA anterior line during the repeat procedure. In this subgroup, LA anterior line was visible on cMRI in all segments in 3 (37%) patients and was not visible in all segments in 5 (63%) patients showing no statistical difference between groups (p = 1.0).

### 3.5. Adverse Events

Acute adverse events during the index hospitalization occurred in four patients (5%). One patient developed cardiac tamponade requiring pericardiocentesis in the contact force information blinded group and could be discharged without any further problems. In this patient, no excessive contact force was recorded. In two patients (one per group), groin vascular access site pseudoaneurysms were treated without necessity for surgical intervention. One patient in the contact force information blinded group developed a groin AV-fistula, which resolved without surgical intervention. No significant difference in adverse event rates was noted between both groups (p=0.61).

## 4. Discussion

In this prospective, randomized clinical trial of patients undergoing PVI for AF, display of contact force information during the application of an additional LA anterior line did not reduce the radiofrequency time to achieve bidirectional block of the line. In addition, contact force, procedural measures, visibility of the LA anterior line on cMRI, and the AF ablation outcome after 12 months of follow-up were similar between both groups.

### 4.1. Contact Force during Linear Ablation

In this study, 72 patients were randomized to application of a LA anterior line either with or without the display of contact force information. In all patients, the same ablation catheters and mapping systems were used. The primary endpoint of time to bidirectional block of the LA anterior line, as well as various secondary endpoints, did not differ significantly between groups. Until now, no study addressing the efficacy and effectiveness of contact force information on LA linear ablations was available. Prior studies using conventional ablation catheters achieved bidirectional block of LA anterior lines in only 60% to 86% [2, 3]. We thus hypothesized that information on contact force could improve the time to achieve bidirectional block of the LA anterior, but failed to identify significant differences. However, our rate of achieved bidirectional block (97%) was much higher than previously reported using conventional ablation catheters. Possible explanations may include longer investigator experience in LA line ablation compared to early reports with limited experience, as well as improvements in ablation catheter technology, even without information on contact force.

In multiple studies, use of contact force for PVI has shown a reduction of acute pulmonary vein reconnection [12–14]. However, in most studies either no randomized protocol was used, or the contact force sensing catheter was compared to a different catheter without contact force sensing capability.

Similar to our study, Ullah et al. randomized 117 patients undergoing PVI with or without information on contact force using the same ablation catheter (Smart-Touch, Biosense Webster) [6]. The primary endpoint was time to achieve complete PVI. In their study, time to achieve PVI, procedure time, and fluoroscopic data did not differ significantly between both groups; in addition, mean contact force was identical in both groups with 13.4g. However, time in contact force target range (5-40g) was significantly higher (80% versus 68%, p<0.001) and the rate of acute PV reconnection was significantly lower (22% versus 32%, p=0.03) in those with versus those without display of contact force data. This is partly in line with our data, where mean contact force and force time integral were comparable between both groups. However, in our cohort, 80% of ablation points reached a target contact force ≥10g, irrespective of randomization. A possible explanation might be different anatomical reachability of the pulmonary veins compared to the LA anterior wall.

### 4.2. Visibility of the Anterior Line on cMRI

Patients underwent cMRI shortly before and three to six months after ablation with the aim of visualizing the LA anterior line [15] and to associate it with the applied contact force. In 81% of cMRIs after ablation, the LA anterior line was visible in at least one segment. However, a visible line in all three segments could only be detected in 52% of patients. This contrasts a bidirectional electrical block that was achievable in 92% of patients. Similarly in a prior study, ablation lines could only be recognized in 54% of patients using cMRI with late gadolinium enhancement [16]. In our cohort, patients without visible lines in all three segments did not show a lower contact force compared to patients with a visible line. This is in contrast to Andreu et al., where gaps in the ablation lines around the pulmonary veins correlated with reduced contact force (6.7 versus 12.2g) in 36 patients [17]. As in our cohort, 80% of ablation points had a contact force ≥10g. Radiofrequency applications with low contact force at the anterior wall were few and a significant difference might have been missed. However, other studies were not able to substantiate a clear correlation between ablation gaps and scar tissue on cMRI either [18]. Such heterogeneity implies that cMRI image acquisition and postprocessing are variable across studies, and further efforts are required to standardize and harmonize cMRI protocols for future studies and to improve lesion imaging.

### 4.3. Success at 12-Month Follow-Up

After systematic 12-month follow-up including repetitive 7-day Holter-monitoring and detailed questioning, we did not find a significant difference in AF recurrence and single procedure success between both groups. This is in line with existing work, where the use of contact force did not improve the rate of sinus rhythm after midterm follow-up [6, 12, 13, 19]. Also, a 1-year success rate of about 50% in patients undergoing ablation for persistent AF appears to be comparable to others [10]. New technologies like ablation index [20] and use of higher contact fore or the use of high power ablation might improve ablation outcome. However, prospective randomized trials on this subject are still under way.

## 5. Limitations

Our results clearly show no relevant differences between our study groups randomized to LA anterior line ablation with or without display of contact force information. It appears unlikely that a larger cohort would have relevantly changed our results. However, we submit that we did not observe the anticipated difference in procedure time that was the basis for our power calculation. It is thus possible that we have missed significant differences due to a lack of statistical power. Complete cMRI data before and after the ablation procedure was only available in 50 patients with no difference between both groups (visible group: 24 versus blinded group: 26, p = 0.45). This might have further reduced our statistical power for cMRI specific analyses. In most of the procedures, we did not use a deflectable sheath for the ablation catheter; perhaps this would have improved the efficacy of the linear ablation. However, 97% of bidirectional block is higher than previously published.

## 6. Conclusion

In this prospective randomized clinical trial, information on contact force during the ablation of a LA anterior line did not reduce radiofrequency time to achieve bidirectional line block. In addition, other procedural data as well as the ablation success rate after 12 months of follow-up did not reveal significant differences between both groups. By cMRI, LA anterior line visibility did not significantly correlate with the applied contact force. In conclusion, information on contact force does not seem to improve procedural and overall ablation success with respect to LA anterior line ablation. In addition, creating anterior line as the only additional lesion in patients with persistent AF in the attempt to improve the success rate of the procedure is still disputable and, therefore, it just remains a partial technique, due to the complexity of the pathophysiology of persistent AF.

## Figures and Tables

**Table 1:** Baseline characteristics.

	**Contact force information visible**	**Contact force information blinded**	**P value**
Age (years)	66±9	62±9	0.06
Sex (male)	24 (65%)	25 (71%)	0.62
AF duration (years)	4.8±3.1	3.7±3.7	0.21
Persistent AF	28 (78%)	32 (91%)	0.19
LA diameter (mm)	43.6±5	43.8±5	0.85
EHRA Score (mean)	2.3±0.9	2.2±0.9	0.87
CHADS Vasc Score (mean)	2.2±1.0	2.2±1.6	0.99
HAS Bled Score (mean)	1.4±0.7	1.1±0.8	0.2
Arterial hypertension (%)	22 (66%)	21 (60%)	0.8
Vascular disease (%)	6 (18%)	7 (20%)	1.0
Heart failure (%)	4 (12%)	6 (17%)	0.47
Previous stroke (%)	2 (6%)	6 (17%)	0.26
Diabetes (%)	2 (6%)	2 (6%)	1.0

*Data are mean±standard deviation or frequency (percentage). LA: left atrial; EHRA: European Heart Rhythm Association*.

**Table 2:** Procedural data.

	**Contact force information visible**	**Contact force information blinded**	**P value**
Procedure time (min)	192.6±30	189±60	0.7
Total fluoroscopic time (min)	23.3±10	27.9±13	0.12
LA anterior line fluoroscopy time (min)	1.1±1.2	0.8±1.1	0.3
Radiation dose (cGy*∗*cm^2^)	1930±2025	1692±1128	0.57
Time to complete LA anterior line (min)	23 ±18	21±15	0.5
LA anterior line reconnection during waiting period	6 (16%)	6 (17%)	0.9
LA anterior line RF applications (n)	10±7	10.4±11	0.9

*Data are mean±standard deviation. LA: left atrial; RF: radiofrequency*.

## Data Availability

The data used to support the findings of this study are available from the corresponding author upon request.
